# *CircPLXNA2* Affects the Proliferation and Apoptosis of Myoblast through *circPLXNA2*/*gga-miR-12207-5P*/*MDM4* Axis

**DOI:** 10.3390/ijms24065459

**Published:** 2023-03-13

**Authors:** Xu Dong, Jiabao Xing, Qingchun Liu, Mao Ye, Zhen Zhou, Yantao Li, Rongqin Huang, Zhenhui Li, Qinghua Nie

**Affiliations:** 1 State Key Laboratory for Conservation and Utilization of Subtropical Agro-Bioresources, Lingnan Guangdong Laboratory of Agriculture, College of Animal Science, South China Agricultural University, Guangzhou 510642, China; 2 Guangdong Provincial Key Lab of Agro-Animal Genomics and Molecular Breeding, and Key Laboratory of Chicken Genetics, Breeding and Reproduction, Ministry of Agriculture, Guangzhou 510642, China; 3 Key Laboratory of Zoonosis Prevention and Control of Guangdong Province, College of Veterinary Medicine, South China Agricultural University, Guangzhou 510642, China; 4 Maoming Branch, Guangdong Laboratory for Lingnan Modern Agriculture, Maoming 525000, China

**Keywords:** myogenesis, apoptosis, *circPLXNA2*, ceRNA, *MDM4*, *p53*

## Abstract

CircRNAs are newly identified special endogenous RNA molecules that covalently close a loop by back-splicing with pre-mRNA. In the cytoplasm, circRNAs would act as molecular sponges to bind with specific miRNA to promote the expression of target genes. However, knowledge of circRNA functional alternation in skeletal myogenesis is still in its infancy. In this study, we identified a circRNA–miRNA–mRNA interaction network in which the axis may be implicated in the progression of chicken primary myoblasts’ (CPMs) myogenesis by multi-omics (i.e., circRNA-seq and ribo-seq). In total, 314 circRNA–miRNA–mRNA regulatory axes containing 66 circRNAs, 70 miRNAs, and 24 mRNAs that may be relevant to myogenesis were collected. With these, the *circPLXNA2*-*gga-miR-12207-5P*-*MDM4* axis aroused our research interest. The *circPLXNA2* is highly differentially expressed during differentiation versus proliferation. It was demonstrated that *circPLXNA2* inhibited the process of apoptosis while at the same time stimulating cell proliferation. Furthermore, we demonstrated that *circPLXNA2* could inhibit the repression of *gga-miR-12207-5p* to *MDM4* by directing binding to *gga-miR-12207-5p*, thereby restoring *MDM4* expression. In conclusion, *circPLXNA2* could function as a competing endogenous RNA (ceRNA) to recover the function of *MDM4* by directing binding to *gga-miR-12207-5p*, thereby regulating the myogenesis.

## 1. Introduction

Chicken is an economically significant domestic animal that plays an indispensable role in human meat consumption as the most consumed food source. China is the largest country for commercial broil farming in the world, with an annual broil slaughter number of 11.83 billion, and the second ranking-country for chicken production, having produced 1878.2 tons in 2021 (http://www.stats.gov.cn/) (accessed on 1 March 2023). According to the USDA, world broiler production will be 10,108.6 million tons in 2022, with a growth rate of 0.57%. The top four ranking countries and regions’ broiler production, i.e., the US, China, Brazil, and the EU are 21.5 million tons, 14.3 million tons, 14.25 million tons, and 10.92 million tons, respectively (https://apps.fas.usda.gov/psdonline/app/index.html#/app/downloads, accessed on 1 March 2023) Due to its economic value, the muscle growth and development of chicken are of great importance and have become the industry’s primary focus. As an essential component of the body, skeletal muscle plays a crucial role in maintaining movement, regulating metabolism, etc. Importantly, the growth and development of skeletal muscle are closely related to meat yield [1]. Multiple studies have demonstrated in recent years that the non-coding RNA (ncRNA) family regulates the growth and development of skeletal muscle to a large extent, due to the genome’s predominance (more than 98%) [2].

Among these non-coding RNA molecules, circRNA is a newly discovered type of endogenous RNA that forms a closed-loop structure when pre-mRNA is covalently spliced into it via back-splicing [2,3]. It is common knowledge that circRNA possesses a distinctive biological structure, and its location determines the multiple regulatory functions it can perform [4]. CircRNA is resistant to RNase R digestion and has a high stability because it lacks a 5′cap and 3′ploy A tail [5]. A CircRNA sequencing platform has been successfully deployed, and a significant amount of circRNA had been characterized since its inception [6,7]. Using this sequencing method, several circRNAs have been identified to regulate the muscle growth process, such as *CircZNF609* and *CircLMO7* [8,9]. However, due to the short time span since the discovery, knowledge of circRNA’s functional alternation in skeletal myogenesis is still in its infancy.

MicroRNA (miRNA) is another class of endogenous non-coding RNA molecules that regulates biological phenotype by target binding the 3′UTR of mRNA. Since the discovery of the very first microRNA in 1993, research into the biological role of miRNA has covered a wide range of topics [10]. Numerous studies have shown that miRNA is very important for controlling the skeletal muscle phenotype. *MiR-27b-3p*, for instance, is suggested to exert a particular effect on muscle atrophy through the *Cbl-b* gene [11]. As another example, *miR-1*, *miR-133*, and *miR-499* have also been reported to regulate myogenesis through binding to the 3′UTR of mRNA [12,13]. However, a particular molecular sponge, known as competing endogenous RNA, may be able to reverse the binding effect (ceRNA, i.e., circRNA).

The *P53* gene is the most inactivated tumor inhibitor gene in cancer [14,15,16]. The function of *p53* may be activated as a result of the heterogeneous pressure in one of two ways: cell cycle arrest or apoptosis [17,18]. Several factors rarely activate the *p53* gene to ensure normal cell cycle under physiological conditions [19]. Murine double minute (MDM) 4, an important p53-binding protein, could interact with *MDM2* to form *MDM2/MDMX* heterodimer to act as an important negative regulator of the *p53* upstream signaling pathway [20]. Numerous studies have demonstrated its biological role in regulating cell growth, but only a few have identified its potential role of co-regulating myogenesis with circRNA [20,21].

In this study, we combined circRNA sequencing and ribosome footprinting to characterize a potential interaction network involving circRNA, miRNA, and mRNA that may regulate muscle development. In this profiling, we discovered a new circRNA called *circPLXNA2*, which we found to have the potential to interact with the molecules *gga-miR-12207-5p* and *MDM4*. We used functional assays to investigate the mechanism of phenotype alternation and potential interactions.

## 2. Results

### 2.1. The circRNA–miRNA–mRNA Axes Network Is Constructed by Multi-Omics

**Figure 1:**
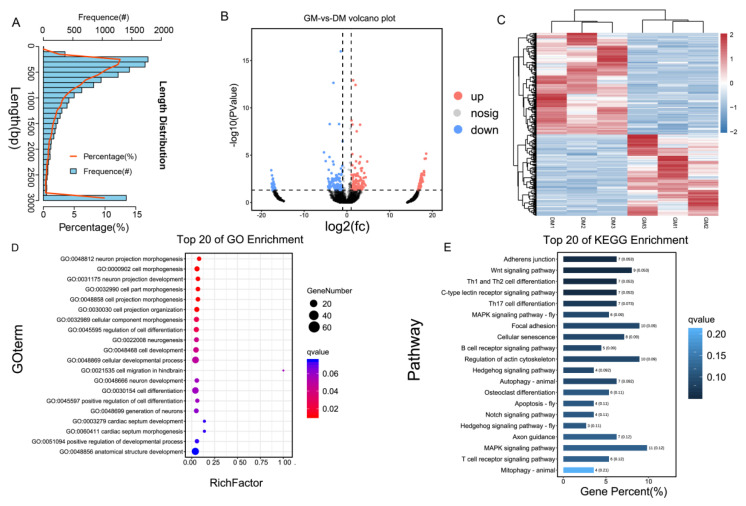
Overview of circular RNA sequencing. (**A**) circRNA length distribution in GM relative to DM. (**B**,**C**) Volcano plot (**B**) and heatmap (**C**) of differentially expressed circRNA between proliferation relative to differentiation in chicken myoblasts. (**D**,**E**). GO functions (**D**) and KEGG pathways. (**E**) Analysis of the parental genes of differentially expressed circRNAs.

**Figure 2:**
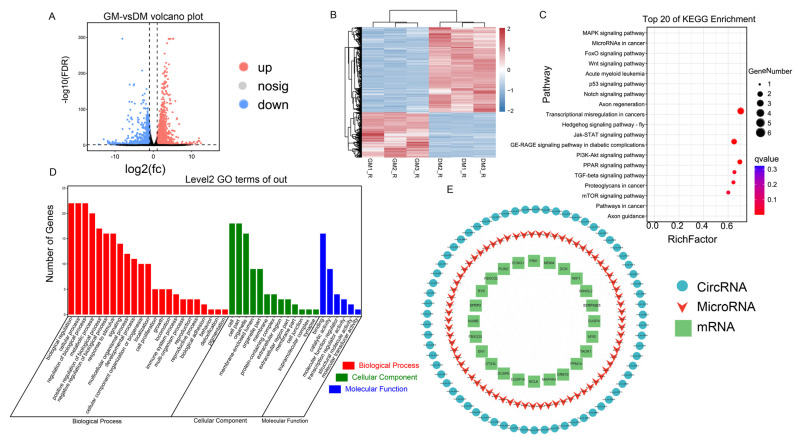
Overview of the ribosomal footprinting and circRNA–miRNA–mRNA interaction network analysis. (**A**,**B**) Volcano plot (**A**) and heatmap (**B**) of differentially expressed mRNAs between GM relative to DM in chicken myoblasts. (**C**,**D**) GO functions (**C**) and KEGG pathways (**D**) analysis of differentially expressed mRNAs. (**E**) The circRNA–miRNA–mRNA regulatory axes network associated with muscle development, with blue circles representing circRNAs, red triangles representing miRNAs, and green rectangles representing mRNAs.

We used circRNA-seq and ribo-seq on chicken primary myoblasts (CPMs) to identify candidate circRNA involved in muscle growth regulation by examining differentially expressed circRNA and regulatory genes. First, circRNA-seq identified 14,241 circRNAs distributed among different chromosomes, with a centralized length span of 0–1000 bp, in which 360 were significantly differentially expressed (Figure 1A–C, Figures S1–S3). After filtration, 200 circRNAs were identified as up-regulating, and 160 circRNAs were identified as down-regulating in differentiation medium (DM) relative to the growth medium (GM) of CPMs (Figure 1B,C, Table S1). We mapped differentially expressed circRNAs to their parental genes and performed GO and KEGG enrichment analysis. GO enrichment analysis suggested a large variety of functional gene enrichment in the process of cellular development and differentiation, and KEGG biological pathway identification indicated that the genes were involved in certain signal pathways, such as MAPK, Wnt, and autophagy (Figure 1D,E). In addition, a total of 3342 differentially expressed genes (DEGs) were identified by ribo-seq profile, in which 2185 DEGs were up-regulated and 1157 DEGs were down-regulated (Figure 2A,B, Figure S4). GO enrichment analysis enriched the most genes into biological processes, such as biological regulation, signaling, and cell proliferation. KEGG signaling pathway analysis indicated that most signaling pathways associated with muscle development were regulated, such as the p53, MAPK, mTOR, and JAK-STAT pathways (Figure 2C,D).

Combined with the newly identified circRNA and mRNA with differentiated abundance, we characterized a circRNA–miRNA–mRNA interaction network that may be involved in regulating the myogenesis process. To be specific, 314 circRNA–miRNA–mRNA regulatory networks with 66 circRNAs, 70 miRNAs, and 24 mRNAs which may be related to myogenesis were acquired as candidate regulation network for further study (Figure 2E, Table S2).

### 2.2. CircPLXNA2 Is a Novel-Identified circRNA Regulated by PLXNA2

**Figure 3:**
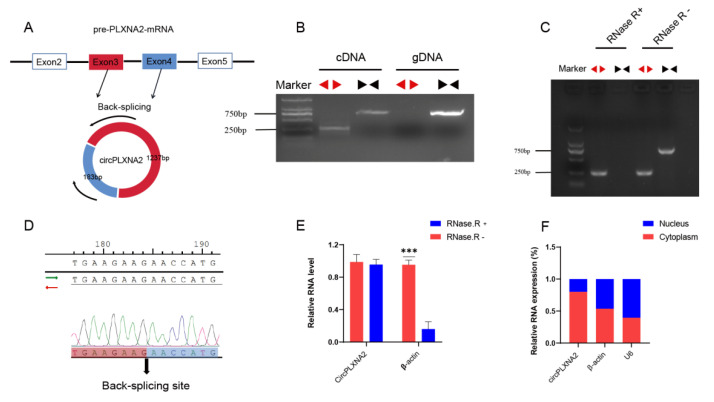
Identification of *circPLXNA2*. (**A**) Schematic diagram of the cyclization of pre-PLXNA2-mRNA. (**B**) The divergent primers could only amplify *circPLXNA2* in cDNA but not in gDNA, as visualized in the agarose gel electrophoresis. Black triangles represent convergent primers and red triangles represent divergent primers. (**C**) Convergent primers failed to amplify the target band with RNase R-treated cDNA, whereas divergent primers could successfully amplify the target bands visualized in the agarose gel electrophoresis. Black triangles represent convergent primers and red triangles represent divergent primers. (**D**) Sanger sequencing confirmed the back-splicing junction sequence of *circPLXNA2*. (**E**) Relative mRNA expression of *circPLXNA2* and β-actin after RNase R treatment. (F) The location of *circPLXNA2* in the cytoplasm and nucleus of CPM was analyzed by qRT-PCR. *GAPDH* and *U6* were served as cytoplasmic and nuclear localization controls, respectively (*** *p* < 0.001).

The circPLXNA2-gga-miR-12207-5P-MDM4 axis was the one that piqued our interest for research purposes among these interaction axes. The circPLXNA2, novel_circ_010465, was termed on the basis of its transcriptional precursor (i.e., pre-PLXNA2-mRNA). Sanger sequencing was initially used to validate the circPLXNA2 back-splicing junction (Figure 3A,D). We then validated the circular structure of circPLXNA2. We designed specific divergent and convergent primers and used genomic DNA (gDNA) and cDNA as templates, respectively, for a PCR reaction to verify the genomic structure. The expected double bands were inclusively shown in the cDNA group visualized by agarose gel electrophoresis, while when we selected gDNA as an amplifying template, the expected band was merely shown in the convergent primers’ lane with weak detection (Figure 3B). In addition, we employed RNase R treatment to detect the resistance of circPLXNA2, which showed successful amplification of circular transcripts by divergent primers using the RNase R-treated cDNA as a template, whereas it failed to be amplified by using convergent primers. In contrast, targeted bands were successfully amplified by both primers using the cDNA template without RNase R digestion (Figure 3C). We also quantified the RNA expression of the circPLXNA2 after RNase R treatment, which showed no significant effect on the expression level of circPLXNA2 after digestion, whereas the expression of β-actin was significantly decreased after digestion (Figure 3E). Together, both findings supported circPLXNA2′s actual existence as a circular structure. Nuclear and cytoplasmic localization experiments of circPLXNA2 were performed to further identify its distribution, and the results showed that circPLXNA2 was present in both the nucleus and the cytoplasm, with its primary localization being in the latter (Figure 3F).

### 2.3. CircPLXNA2 Promotes Cell Proliferation and Inhibits Cell Apoptosis

**Figure 4:**
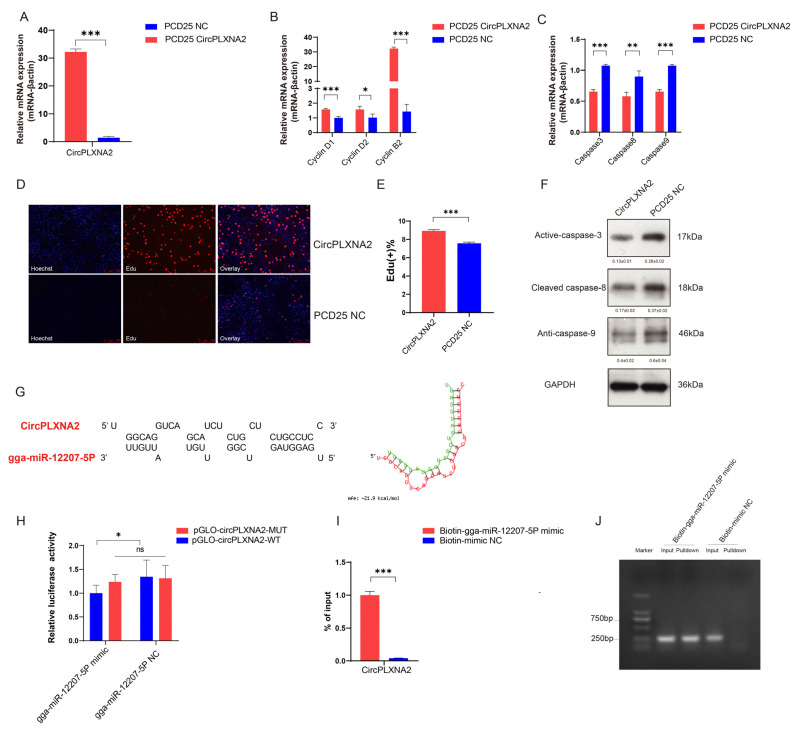
*CircPLXNA2* promoted cell proliferation, inhibited cell apoptosis, and sponged gga-miR-12207-5P. (**A**) Efficiency of overexpression of *circPLXNA2*. (**B**) qRT-PCR results of Cyclin B2, cyclin D1, cyclin D2, and cyclin B2 genes after *circPLXNA2* overexpression. (**C**) qRT-PCR results of caspase 3, caspase 8, and caspase 9 genes after *circPLXNA2* overexpression. (**D**,**E**) EdU staining (**D**) and the positive EdU cell rate (E) for myoblast cells after *circPLXNA2* overexpression. (**F**) Western blotting analysis of the caspase 3, caspase 8, and caspase 9 proteins with PCD25 *circPLXNA2* transfection. (**G**) Potential binding sites between *circPLXNA2* and gga-miR-12207-5P predicted by the RNAhybrid online tool. (**H**) Relative luciferase activity of pmirGLO-*circPLXNA2* WT/MUT plasmid with gga-miR-12207-5P mimic or gga-miR-12207-5P NC. (**I**,**J**) The biotin-tagged miRNA pulldown showed the interaction between *circPLXNA2* and gga-miR-12207-5P (* *p* < 0.05; ** *p* < 0.01, *** *p* < 0.001).

To identify the potential biological function of *circPLXNA2* in myogenesis, we constructed the overexpression vector of *circPLXNA2*. As shown in Figure 4A, the relative mRNA expression level of *circPLXNA2* was drastically increased after transfecting PCD25 *circPLXNA2* (Figure 4A). Overexpression of *circPLXNA2* in chicken primary myoblasts (CPMs) increased the relative expression of proliferation biomarker genes (*cyclin D1*, *cyclin D2*, and *cyclin B2*), suggesting that it may contribute to proliferation (Figure 4B). Furthermore, we detected the relative expression level of apoptosis-related genes, which revealed that overexpression of *circPLXNA2* suppressed the relative expression of caspase 3, caspase 8, and caspase 9 at the mRNA level, indicating detectable apoptosis suppression by *circPLXNA2* (Figure 4C,F). In addition, more 5-ethynyl-2’-deoxyuridine (EdU)-stained cells were found in the *circPLXNA2* overexpression group than in the control group (PDC25 negative Control, PDC25 NC) (Figure 4D,E). These findings together suggested that *circPLXNA2* could control the development of CPMs by regulating cell proliferation and apoptosis. As we predicted an interaction network involving miRNA (i.e., gga-miR-12207-5P), we hypothesized that *circPLXNA2* could serve as an endogenous sponge to competitively interact with gga-miR-12207-5P, thereby altering the distribution of gga-miR-12207-5P against their target. The potential binding site between circPLXNA2 and gga-miR-12207-5P is predicted by the RNAhybrid online tool (Figure 4G) (https://bibiserv.cebitec.uni-bielefeld.de/rnahybrid) (accessed on 1 November 2022). We used the dual-luciferase reporter assay and the pulldown assay to characterize their potential interaction. Compared with the gga-miR-12207-5P mimic and Pmir-GLO-*circPLXNA2*-MUT co-transfection group, gga-miR-12207-5P mimic co-transfected with the Pmir-GLO-*circPLXNA2*-WT vector significantly repressed the relative luciferase activity (Figure 4H). Gel electrophoresis and biotin-labelled miRNA pulldown analysis showed that biotin-gga-miR-12207-5P mimic transfection significantly enriched circPLXNA2 mRNA levels in CPMs (Figure 4I,J). Collectively, these findings suggested that *circPLXNA2* and gga-miR-12207-5P have a targeted binding relationship.

### 2.4. gga-miR-12207-5P Inhibits Cell Proliferation and Promotes Apoptosis

**Figure 5:**
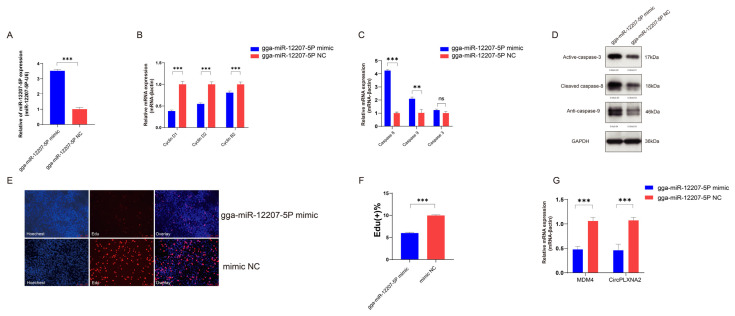
gga-miR-12207-5P inhibited cell proliferation and promoted cell apoptosis. (**A**) The overexpression efficiency of gga-miR-12207-5P in myoblasts after transfection with gga-miR-12207-5P mimic. (**B**) qRT-PCR results of cyclin D1, cyclin D2, and cyclin B2 after gga-miR-12207-5P overexpression. (**C**,**D**) mRNA level (**C**) and translation level (**D**) of caspase 3, caspase 8, and caspase 9 after gga-miR-12207-5P overexpression. (**E**,**F**) EdU staining (**E**) and the positive EdU cell rate (**F**) for myoblast cells after gga-miR-12207-5P overexpression. (**G**) The expression levels of *circPLXNA2* and *MDM4* were significantly decreased in myoblasts transfected with gga-miR-12207-5P mimic (** *p* < 0.01, *** *p* < 0.001, and ns: no significance).

To explore the biological function of gga-miR-12207-5P, we designed the overexpression vector of gga-miR-12207-5P and termed it the gga-miR-12207-5P mimic. After 48 h of transfection, the relative expression level of gga-miR-12207-5P was significantly higher than the control group, indicating reliable overexpression efficiency. (Figure 5A). After transferring gga-miR-12207-5P mimic into CPMs 48 h, cyclin D1, cyclin D2, and cyclin B2 were all significantly downregulated compared to the mimic negative control (mimic NC) treatment, suggesting that it inhibits proliferation (Figure 5B). After 48 h of transfection, the apoptosis pathway was activated, as evidenced by an increase in the relative expression level of caspase 8, caspase 9, and caspase 3 (Figure 5C,D). EdU staining further supported the fact that gga-miR-12207-5P overexpression significantly repressed the viability of CPMs (Figure 5E,F). Overall, the viability of CPMs may be downregulated by overexpression of gga-miR-12207-5P.

Additionally, we examined the potential interaction between gga-miR-12207-5P, *MDM4*, and *circPLXNA2*. Overexpression of gga-miR-12207-5P decreased *MDM4* and *circPLXNA2* mRNA abundance, suggesting that it inhibits their transcriptional activity (Figure 5G).

### 2.5. MDM4 Promotes Cell Proliferation and Inhibits Cell Apoptosis through the P53 Signaling Pathway

**Figure 6:**
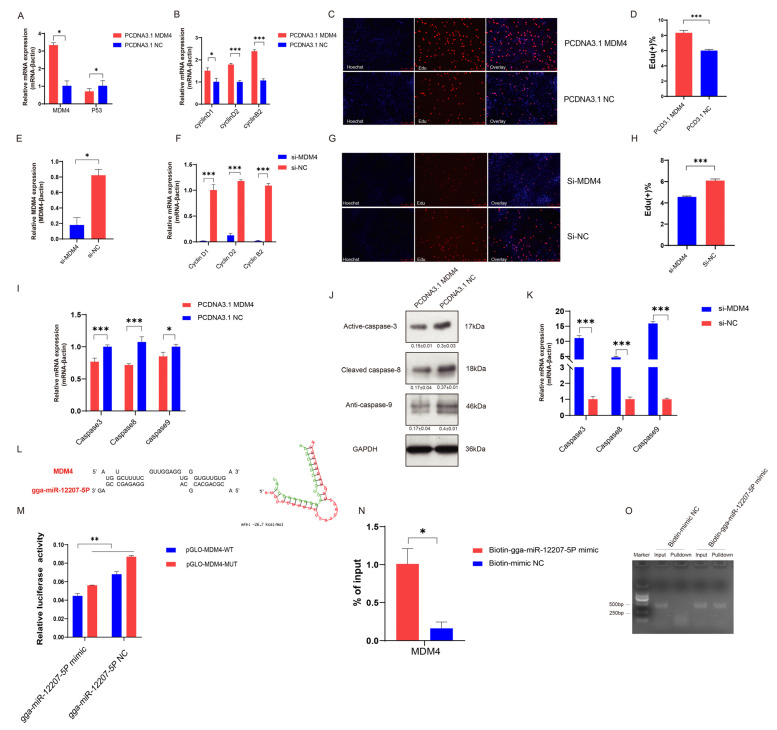
*MDM4* promotes cell proliferation and inhibits apoptosis through the P53 signaling pathway. (**A**) *MDM4* and p53 mRNA levels after transfection with *MDM4* overexpression plasmid in CPMs. (**B**) qRT-PCR results of cyclin D1, cyclin D2, and cyclin B2 after *MDM4* overexpression. (**C**,**D**) EdU staining (**C**) and the positive EdU cell rate (**D**) for myoblast cells after *MDM4* overexpression. (**E**) *MDM4* mRNA levels after transfection with *MDM4* silencing vector in CPMs. (**F**) qRT-PCR results of cyclin D1, cyclin D2, and cyclin B2 after *MDM4* silencing. (**G**,**H**) EdU staining (**G**) and the positive EdU cell rate (H) for myoblast cells after *MDM4* knockdown. (**I**,**J**) Relative mRNA level (**I**) and protein level (**J**) of caspase 3, caspase 8, and caspase 9 expression after *MDM4* overexpression. (**K**) Relative mRNA level expression of caspase 3, caspase 8, and caspase 9 genes after *MDM4* gene silence. (**L**) Potential binding sites between *MDM4* and gga-miR-12207-5P predicted by RNAhybrid online tool. (**M**) Relative luciferase activity of pmirGLO-*MDM4* WT/MUT plasmid with gga-miR-12207-5P mimic or gga-miR-12207-5P NC. (**N**,**O**) mRNA level (**N**) and RNA level (**O**) of the biotin-tagged miRNA pulldown assay (* *p* < 0.05; ** *p* < 0.01, *** *p* < 0.001).

To confirm *MDM4*′s biological function in the myogenesis process, we created an overexpression plasmid of *MDM4* to detect it and transfected this plasmid into CPMs, which showed a significant repression of the relative mRNA expression of *p53*, a critical signaling pathway regulating cell apoptosis, indicating that *MDM4* may inhibit myogenesis via apoptosis. (Figure 6A). As such, we detected the relative expression level of *cyclin D1*, *cyclin D2*, and *cyclin D3* when *MDM4* was overexpressed in CPMs. The results revealed that these genes were significantly promoting expression at the mRNA level.

EdU staining assays also suggested that *MDM4* overexpression induced CPM proliferation (Figure 6B–D). On the contrary, silencing the *MDM4* gene repressed the relative mRNA expression of *cyclin D1*, *cyclin D2*, *and cyclin D3*, as well as the total number of stained cells (Figure 6E–H). Furthermore, we detected the relative expression level of apoptosis-related genes and found that *caspase 3*, *caspase 8*, and *caspase 9* were significantly downregulated 48 h after transfecting an overexpression *MDM4* plasmid (Figure 6I). Western blotting analysis also showed a decreasing trend of *caspase 3*, *caspase 8*, and *caspase 9* after *MDM4* overexpression (Figure 6J). Conversely, we observed the opposite effect of *caspase 3*, *caspase 8*, and *caspase 9* as the expression of *MDM4* was interfered with (Figure 6K). Congruously, these results supported the biological role of *MDM4* in promoting cell proliferation and inhibiting apoptosis. We used the RNAhybrid tool to predict the target binding region of the 3′UTR of *MDM4* and the *gga-miR-12207-5P* seed sequence and identified the best-matched region possessing the least free energy (Figure 6L) (https://bibiserv.cebitec.uni-bielefeld.de/rnahybrid). Subsequently, dual-luciferase reporter vectors (i.e., Pmir-GLO-*MDM4*-WT and Pmir-GLO-*MDM4*-MUT) were constructed and transferred into DF-1 cells to further explore the potential interaction of *MDM4* and *gga-miR-12207-5P*. RT-qPCR results demonstrated the targeted binding relationship between gga-miR-12207-5P and *MDM4* (Figure 6M), which was further confirmed by a biotin-coupled miRNA pulldown assay (Figure 6N,O).

### 2.6. CircPLXNA2 Affects the Proliferation and Apoptosis of Myoblasts by Regulating MDM4 through the Competitive Sponge of gga-miR-12207-5P

**Figure 7:**
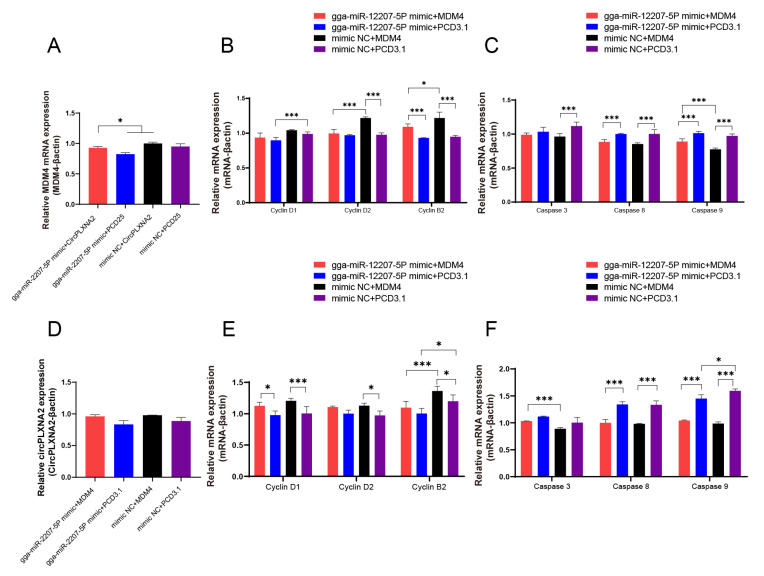
*CircPLXNA2* regulates *MDM4* by competitive adsorption of gga-miR-12207-5P to affect the proliferation and apoptosis of myoblasts. (**A**) The mRNA expression level of *MDM4* after co-transfection of gga-miR-12207-5P mimic and *circPLXNA2* in myoblasts. (**B**) The mRNA expression levels of cyclin D1, cyclin D2, and cyclin B2 after co-transfection of gga-miR-12207-5P mimic and *circPLXNA2* in myoblasts. (**C**) The mRNA expression levels of caspase 3, caspase 8, and caspase 9 after co-transfection of gga-miR-12207-5P mimic and *circPLXNA2* in myoblasts. (**D**) Expression level of *circPLXNA2* after co-transfection of gga-miR-12207-5P mimic and *MDM4* in myoblasts. (**E**) qRT-PCR results of cyclin D1, cyclin D2, and cyclin B2 after gga-miR-12207-5P mimic and *MDM4* co-transfection. (**F**) qRT-PCR results of caspase 3, caspase 8, and caspase 9 after gga-miR-12207-5P mimic and *MDM4* co-transfection. (* *p* < 0.05 and *** *p* < 0.001).

To further characterize the potential interaction relationship between *circPLXNA2*, gga-miR-12207-5P, and *MDM4*, we measured *MDM4* expression levels after overexpressing both gga-miR-12207-5P mimic and *circPLXNA2*. To be more specific, compared to the gga-miR-12207-5P mimic and *circPLXNA2* co-transfection groups, the mRNA expression level of *MDM4* was significantly upregulated in the gga-miR-12207-5P mimic and PCD25 co-transfection groups, as well as the mimic NC and *circPLXNA2* co-transfection groups. Furthermore, the relative mRNA expression level of *MDM4* was lowest in the group that had both gga-miR-12207-5P mimic and PCD25 co-transfected. In contrast, the expression level of *MDM4* was highest when *circPLXNA2* was overexpressed, which suggests that *circPLXNA2* may reverse the effect of gga-miR-12207-5P on *MDM4* (Figure 7A).

We further quantified the cyclin B2, cyclin D1, and cyclin D2 genes after overexpressing gga-miR-12207-5P and *circPLXNA2* in CPMs, individually or in combination, to confirm the potential proliferation effect of both molecules. As shown, the RT-qPCR results indicated that as *circPLXNA2* was overexpressed independently, the mRNA relative expression of the three genes was drastically higher than other groups (*p* < 0.05), whereas gga-miR-12207-5P independent overexpression reduced the mRNA relative expression of the three genes. We, therefore, co-transfected *circPLXNA2* and gga-miR-12207-5P mimic and discovered that the activity levels of these three genes were partially restored (Figure 7B). Furthermore, we measured the abundance of the apoptosis markers caspase 3, caspase 8, and caspase 9 mRNAs after overexpression of gga-miR-12207-5P and *circPLXNA2*, respectively, or co-overexpression of both. RT-qPCR measurements showed that when *circPLXNA2* was overexpressed alone, the three apoptosis markers were drastically downregulated compared to the other three groups. However, when we transfected the gga-miR-12207-5P mimic alone, the mRNA abundance of the three markers increased significantly (*p* < 0.05). Subsequently, we co-transfected both gga-miR-12207-5P mimic and *circPLXNA2*, and caspase 3, caspase 8, and caspase 9 were reverted between transfections. (Figure 7C). These findings led us to hypothesize that *circPLXNA2* could free *MDM4* by competing with it for binding to gga-miR-12207-5P, restoring *MDM4*′s ability to promote cell proliferation while simultaneously inhibiting apoptosis. In order to measure the levels of *circPLXNA2*, we co-transfected gga-miR-12207-5P mimic and *MDM4*. We discovered a similar tendency when compared to the *MDM4* expression levels as we transfected gga-miR-12207-5P mimic and *circPLXNA2* separately or both synchronously. We also measured the relative levels of proliferation and apoptosis markers after gga-miR-12207-5P and *circPLXNA2* co-transfection or independent transfection, which showed a close variable trend (Figure 7D–F). Overall, these results back our hypothesis, that is, *circPLXNA2* recovered the function of *MDM4* by competitively binding to gga-miR-12207-5P, thereby recovering the promotion of cell proliferation and inhibition of apoptosis by *MDM4*.

## 3. Discussion

CircRNAs are new members of the non-coding RNA family that emerged from the backbone of pre-mRNA back splicing and were previously classified as “transcriptional noise” in mammals [22,23]. Furthermore, the conventional mode of study is inapplicable due to the circRNA’s low abundance and expressive quantity [24]. However, with the progress of the genome sequencing approach, circRNAs are increasingly recognized to be implicated in regulating disease phenotypes. Depending on their subcellular location, circRNAs are regulated in two distinct ways [25]. In the nucleus, circRNAs combine with RNA polymerase II to regulate the transcriptional activity [26]. Alternatively, in the cytoplasm, circRNAs act as molecular sponges to bind with specific miRNA to promote the function of target genes [27,28,29,30,31,32]. For example, it has been demonstrated that circPTPN4 can stimulate NAMPT expression by sponging miR-499-3P in the cytoplasm and acting as a competing endogenous RNA [13]. Hence, here we favor a model to identify the circRNA–miRNA–mRNA interaction network in which the axes may be implicated in the progress of CPMs myogenesis in a combination of multi-omics (i.e., circRNA-seq and ribo-seq). To be more specific, we first used circRNA-seq to identify a total of 360 circRNAs with differential expression during the GM compared to the DM. We further presented the 3342 ribo-seq-identified differentially-transcribed genes, thereby selecting the genes most likely to play a role in myogenesis during the GM as opposed to the DM. Combining these, we characterized a circRNA–miRNA–mRNA network that may participate in regulating myogenesis.

In our study, we identified a novel circRNA with heterogeneous expression in GM relative to DM and termed *circPLXNA2*, which is generated from the 3-4 exon of the PLXNA2 gene. Overexpression, real-time quantitative PCR, a dual-luciferase reporter assay, RNA pulldown, and the addition of EdU were the methods that we used to demonstrate the potential biological role of *circPLXNA2*. We found that *circPLXNA2* could promote the relative mRNA abundance of cyclin D1, cyclin D2, and cyclin D3 and inhibit mRNA abundance of caspase 3, caspase 8, and caspase 9, facilitating cell proliferation and repressing apoptosis. In addition, the interaction between gga-miR-12207-5P and *circPLXNA2* was investigated further and found to be consistent with the results of our in-silico analysis.

**Figure 8:**
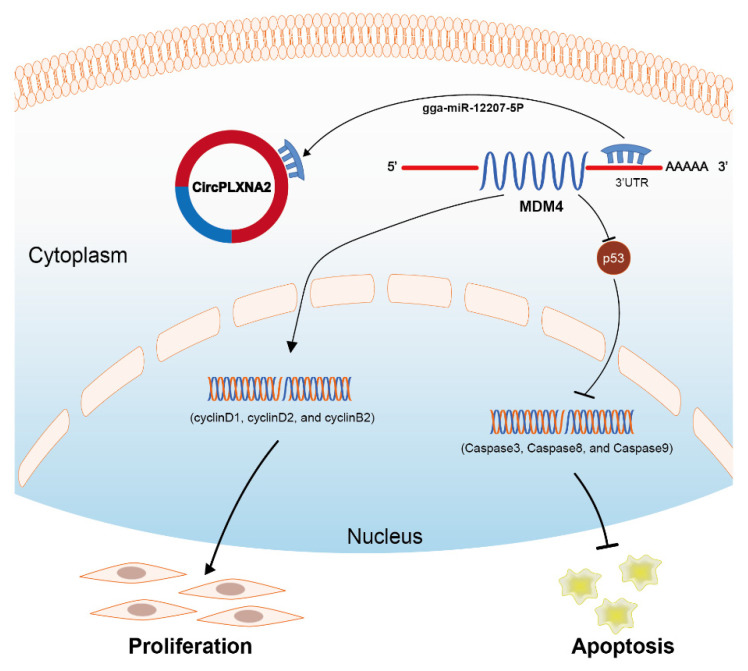
Schematic diagram of how *CircPLXNA2* affected the proliferation and apoptosis of the myoblast *circPLXNA2*/gga-miR-12207-5P/*MDM4* axis.

*MDM4* is constituted as a p53 inhibitor to regulate p53 activity, thereby inhibiting cell apoptosis and promoting proliferation [20]. P53 is a sensor of genotoxic stress that could protect cells from DNA damage by inducing cell-cycle arrest [21]. Numerous studies have shown that p53 induces apoptosis via both transcriptional activation of pro-apoptotic and repression of anti-apoptotic genes, as well as non-transcriptional mechanisms [14,15]. As exemplified by Cai et al., miR-16-5p could inhibit proliferation and promote apoptosis using the p53 signaling pathway by regulating SESN1 mRNA relative expression [33]. Here, we showed that gga-miR-12207-5P has the opposite effect on *circPLXNA2*, namely, that it can suppress *MDM4*, which controls myoblast proliferation and apoptosis via the p53 signaling pathway (Figure 8). By simultaneously overexpressing *circPLXNA2* and gga-miR-12207-5P, we restored the relative abundance of MDM4, which reversed proliferation and inhibited apoptosis.

## 4. Materials and Methods

### 4.1. Animals and Cell

Thigh muscles of 11 embryo-aged chickens were isolated on sterile forceps using autoclaved surgical equipment and then completely minced. The crushed muscle tissue was digested in a constant temperature incubator at 37 °C for 15 min using 0.25% trypsin-EDTA (Gibco, Carlsbad, CA, USA). Cells were treated with high glucose Dulbecco’s modified Eagle medium (Gibico) containing fetal bovine serum (FBS)10% (*v/v*) and 0.2% penicillin/streptomycin) to terminate digestion. The digested suspension was then filtered through a 70 μm strainer and centrifuged at 1000× *g* for 5 min. The precipitated cells were seeded uniformly in cell culture dishes by blowing with complete medium, and adherent purification was performed twice for 40 min at 37 °C in an incubator with 5% CO_2_ to eliminate the interference of chick embryo fibroblasts. Finally, the medium containing CPM cells was uniformly seeded in cell culture dishes for subsequent experiments.

We incorporated different mediums to prompt cell to reach specific life cycles. For myogenic proliferation, cells were treated with growth medium, which is composed of high glucose Dulbecco’s modified Eagle medium (Gibico) containing FBS 10% (*v/v*) and 0.2% penicillin/streptomycin. For myogenic differentiation, the growth medium was replaced with differentiation medium (RPMI-1640 medium (Gibico) with 2% horse serum) when myoblasts reached 90% cell confluence.

### 4.2. Parallel Generation of circRNA and riboRNA Libraries

We prepared the CPM samples at growth medium (GM) and differentiation medium (DM) for three biological replicates with good cell viability, respectively. Each sample was individually pooled for subsequent RNA extraction and library construction. Total RNA was extracted using TRI-zol^®^ Reagent (Invitrogen, CA, USA) according to the manufacturers’ instruction. Subsequently, the total RNA was treated with RNase R to degrade the linear RNAs and purified using a RNeasy MinElute Cleanup Kit (Qiagen, Hilden, Germany). Genomic DNA was then removed using DNase I (Takara, Dalian, China). CircRNA libraries were constructed using the TruSeqTM Stranded Total RNA Sample Preparation Kit from Illumina (Illumina, Los Angles, USA) after the removal of host ribosomal RNA using Illumina Ribo-ZeroTM rRNA Removal Kits (Qiagen) and RNase R treatment. Next, a strand-specific library was constructed using a VAHTS Total RNA-seq (H/M/R) Library Prep Kit (Vazyme, Nanjing, China) from Illumina, following the manufacturer’s instructions. Briefly, ribosome RNAs were removed to retain circRNAs. The enriched circRNAs were fragmented into short fragments by using fragmentation buffer and reverse transcribed into cDNA with random primers. Second-strand cDNA were synthesized by DNA polymerase I, RNase H, dNTP (dUTP instead of dTTP), and buffer. Next, the cDNA fragments were purified with VAHTSTM DNA Clean Beads (Vazyme), end repaired, poly(A) was added, and then they were ligated to Illumina sequencing adapters. Then, UNG (Uracil-N-Glycosylase) was used to digest the second-strand cDNA. The digested products were purified with VAHTSTM DNA Clean Beads, PCR amplified, and sequenced using Illumina Novaseq 6000 (Illumina).

### 4.3. Ribosome Footprinting

Analogously, we prepared the CPMs during GM and DM three times as biological replicates. The ribosome footprints were generated after immunoprecipitation of cardiomyocyte-specific monosomes with anti-HA magnetic beads after treating the lysate with RNase I. Then, the libraries were constructed according to the manufacturer’s instruction using a mammalian ribo-seq kit (Illumina) with each replicate. Then, the libraries were sequenced using Illumina Novaseq 6000 (Illumina).

### 4.4. CircRNA Validation

We first validated the biological structure of *circPLXNA2* using convergent and divergent primers. Then, the junction sequence of the *circPLXNA2* was sequenced by Tsingke Biotechnology Co., Ltd. (Beijing, China). Furthermore, we detected the RNase R sensitivity using RT-qPCR.

### 4.5. Primers

All primers for this study were designed in Premier Primer v5.0 (Premier Biosoft International, San Francisco, CA, USA), and synthesized by Tsingke Biotechnology Co., Ltd. (Beijing, China). The detailed information on all primers is listed in Table 1.

### 4.6. RNA Oligonucleotides and Plasmids Construction

Gga-miR-12207-5P mimic, mimic NC; si-*MDM4* and siRNA negative control were designed and synthesized by RiboBio (Guangzhou, China). The detailed information of sequences is listed in Table 2.

Construction of the *MDM4* overexpression vector was as follows. The full-length sequence of *MDM4* was amplified by conventional PCR primers, and the amplified sequence fragment was cloned into the commercial vector pcDNA3.1 (Promega, Madison, WI, USA) through the restriction site of HindIII and XhoII.

Construction of the *circPLXNA2* overexpression vector was as follows. First, the full-length linear sequence of *circPLXNA2* was synthesized according to the sequencing results. Subsequently, the EcoRI restriction site, forward looping mediate sequence, and AG receptor were added into the 5′ end of the forward primer. Similarly, the BamHI restriction site, reverse cyclization mediating sequence, and GT donor were added into the 5′ end of the reverse primer. A homology arm was then added to the full-length linear sequence of *circPLXNA2* by PCR reaction, and the fragment was cloned into the circular vector pCD25-ciR by homologous recombinase (Geneseed Biotech, Guangzhou, China).

Construction of PmirGLO dual-luciferase target reporter vector was as follows. The DNA templates of circPLXNA2, MDM4 WT, and circPLXNA2,MDM4 MUT were synthesized by Tsingke Bio-technology Co., Ltd. (Beijing, China). The circPLXNA2 and MDM4 3’UTR segments containing the putative gga-miR-12207-5P binding sequence were then subcloned into the XhoI and SalI restriction sites of the pmirGLO dual-luciferase reporter vector. (Promega, USA) (Table S3).

### 4.7. Cell Transfection

All cell transient transfections in this study were performed after cells grew to 60–80% confluence according to the manufacturer’s protocol of Lipofectamine 3000 reagent (Invitrogen). The nucleic acids were also diluted using OPTI-MEM medium (Gibco). For miRNA mimic, mimic NC, and siRNA transfection, the final transfection concentration was 100 nM.

### 4.8. Dual-Luciferase Reporter Assay

In order to verify the binding relationship between gga-miR-12207-5P and *circPLXNA2* and *MDM4*, we designed the plasmids for several groups of co-transfection experiments, i.e., a. gga-miR-12207-5P mimic+Pmir-GLO-*circPLXNA2*-WT, b. mimic NC+Pmir-GLO-*circPLXNA2*-WT, c. gga-miR-12207-5P mimic+Pmir-GLO-*circPLXNA2*-MUT, d. mimic NC+Pmir-GLO-*circPLXNA2*-MUT, e. gga-miR-12207-5P mimic+Pmir-GLO-*MDM4*-WT, f. mimic NC+Pmir-GLO-*MDM4*-WT, g. gga-miR-12207-5P mimic+Pmir-GLO-*MDM4*-MUT, and h. mimic NC+Pmir-GLO-*MDM4*-MUT were transfected into DF-1 cell lines with 60–80% confluence (96-well plate). At 48 h post-transfection, luciferase activity analysis was examined using a fluorescence/multi-detection microplate reader (BioTek, Winooski, VT, USA) and Dual-GLO^®^ Luciferase Assay System Kit (Promega). Firefly luciferase activities were normalized to Renilla luminescence in each well.

### 4.9. 5-Ethynyl-20-Deoxyuridine (EdU) Assays

To investigate the proliferation of the cells, 50 μM 5-ethynyl-20-deoxyuridine was first added into cells to incubate for 2 h after 36 h of transfection (EdU) (RiboBio, China), with fixing with 4% paraformaldehyde (PFA) for 30 min and neutralizing with 4% PFA with 2 mg/mL glycine; then, they were permeabilized with 0.5% Triton X-100. Cells were subsequently stained using the C10310 EdU Apollo In Vitro Imaging Kit (RiboBio). Finally, fluorescence microscopy (DMi8; Leica Microsystems, Wetzlar, Germany) was used to obtain cell images. At least three random fields were selected for each well to photograph, and Image J was used to count the cells; hence, the cell proliferation rate was calculated.

### 4.10. Biotin-Coupled miRNA Pull Down Assay

When myoblasts in two 10 cm dishes reached 60–80% confluence, 3′end biotin-labelled gga-miR-12207-5P mimic or mimic NC (100 nM, *p* < 0.05) were transfected into the cells alternatively. Cells were collected and washed with PBS after 36 h transfection. Using a miRNA pulldown kit (BersinBio, Guangzhou, China), the lysed cells were incubated with the sealed magnetic beads for 4 h at 4 °C with mild rotation (20 rpm/min) to pulldown the biotin-coupled RNA complex. The RNA was eluted and precipitated as required to obtain the RNA for subsequent RT-qPCR that specifically interacted with gga-miR-12207-5P.

### 4.11. RNA Extraction, cDNA Synthesis and Quantitative Real-Time PCR

As described, total RNA was extracted from tissues or cells using TRIzol reagent^®^ (Invitrogen) according to the manufacturer’s requirements. The concentration and integrity of extracted RNA were tested using NanoDrop One and 1% gel electrophoresis. Then, the cDNA was synthesized using PrimeScript RT Reagent Kit with gDNA Eraser (perfect real-time) (TaKaRa). Real-time quantitative PCR was performed as previously described [13,33,34].

### 4.12. Western Blotting Assay

The cells were incubated with RIPA buffer containing protease inhibitors (protease inhibitors: RIPA = 1:100, Solarbio, Guangzhou, China) for 15 min on ice to fully lyse the cells 48 h after transfection. Then, the cells were centrifuged at 12,000× *g* for 10 min at 4 °C, and the supernatant was removed. Total protein was then quantified using the BCA Protein Assay Kit (Beyotime, Shanghai, China). Proteins were separated in 12% SDS-PAGE and transferred to nitrocellulose membranes (Whatman, Maidstone, UK), blocked with 5% skim milk powder for 1 h, and then incubated with primary antibody solution overnight at 4 °C. Subsequently, PVDF membranes were washed three times for 5 min with TBST solution (Beyotime) and then incubated with secondary antibody solution for 60 min at room temperature. Western immunoblotting results were analyzed using the Odyssey Fc system (LI-COR, Lincoln, NE, USA). Antibody information is as follows: actived-caspase-3 p17 polyclonal antibody (BS7004; Bioworld, MN, USA; 1: 500) cleaved caspase-8 (Asp391) (18C8) rabbit mAb (9496; Cell Signaling Technology, Danvers, MA, USA; 1:1000), anti-caspase-9 antibody [E23] (ab32539; Abcam, Cambridge, UK; 1:1000), rabbit anti-GAPDH (AB-P-R 001; Hangzhou Goodhere Biotechnology Co., Ltd., Hangzhou, China; 1:1500).

### 4.13. Statistical Analysis

In this study, all results were showed as mean ± S.E.M with 3–6 independent replications. An independent sample t-test was used to test the statistically significant difference between groups. We considered *p* < 0.05 to be statistically significant. Here, * *p* < 0.05; ** *p* < 0.01; *** *p* < 0.001.

## 5. Conclusions

In conclusion, our study provided a comprehensive profiling of interaction axes which may regulate the process of myogenesis involving circRNA, miRNA, and mRNA. With these, we proved that a novel circRNA, *circPLXNA2*, is highly heterogeneously expressed in DM relative to GM. Mechanistically, *circPLXNA2* could function as ceRNA to sponge gga-miR-12207-5P to release *MDM4*, a negative regulator of the p53 signaling pathway, thereby exerting myoblast proliferation and repressing apoptosis. Overall, this study showed the first evidence of *circPLXNA2* of regulating skeletal myogenesis, and the *circPLXNA2*-gga-miR-12207-5p-*MDM4* axis may become a potential therapeutic target for muscle development.

## Figures and Tables

**Table 1:** Primers used in this study.

Gene Name	Primer Sequences (5′–3′)	Size (bp)	Application
*CircPLXNA2*	F:CAATGGCTACAGCGTGGTG R:GGTCAGGTGGTTGAAAGTCC	266	Divergent primer and miRNA pulldown
PLXNA2	F:CTGACCCTCACCAACAACG R:AGCACGAATCGGGAAGA	765	Convergent primer
q-*circPLXNA2*	F:CTACAGCGTGGTGTTCGT R:CTTCTTCACTTTGCCATTTT	46	qRT-PCR
q-*MDM4*	F:ACAGTGATGAAGGGATAGA R:CAATAGAGCCACAGGGA	120	qRT-PCR
P-*MDM4*-3′UTR	F:GAGGCTAAATGAGGTGTA R:TGAGATGTCAGCAGGTT	443	miRNA pulldown
*Cyclin D1*	F:CAGAAGTGCGAAGAGGAAGT R:CTGATGGAGTTGTCGGTGTA	188	qRT-PCR
*Cyclin D2*	F:AACTTGCTCTACGACGACC R:TTCACAGACCTCCAACATC	150	qRT-PCR
*Cyclin B2*	F:CAGTAAAGGCTACGAAAG R:ACATCCATAGGGACAGG	133	qRT-PCR
*P53*	F:GAGATGCTGAAGGAGATCAATGAG R:GTGGTCAGTCCGAGCCTTTT	145	qRT-PCR
*Caspase 3*	F:TGGCCCTCTTGAACTGAAAG R:TCCACTGTCTGCTTCAATACC	139	qRT-PCR
*Caspase 8*	F:CCCTGAAGACAGTGCCATTT R:GGGTCGGCTGGTCATTTTAT	106	qRT-PCR
*Caspase 9*	F:TCCCGGGCTGTTTCAACTT R:CCTCATCTTGCAGCTTGTGC	207	qRT-PCR
*β-actin*	F:GATATTGCTGCGCTCGTTG R:TTCAGGGTCAGGATACCTCTTT	194	qRT-PCR

**Table 2:** Oligonucleotide sequences in this study.

Name	Sequences (5′–3′)
si-*MDM4*	AGACTAACCAGGACATAGA
*gga-miR-12207-5P mimic*	ACGCAGCACGCAGGAGAGCCGAG

## Data Availability

The circRNA-seq and the ribo-seq profiles have been deposited in the China National GeneBank DataBase (CNGBdb) under the accession number CNP0003882. Any remaining data could be acquired in the supplement files or requested to the corresponding author.

## Supplementary Materials

The following supporting information can be downloaded at: https://www.mdpi.com/article/10.3390/ijms24065459/s1.
