# Supplementary material for: CircPLXNA2 Affects the Proliferation and Apoptosis of Myoblast through circPLXNA2/gga-miR-12207-5P/MDM4 Axis
Source: Int J Mol Sci. 2023 Mar 13;24(6):5459. doi: 10.3390/ijms24065459 (PMC10049439; doi:10.3390/ijms24065459)
Supplement: Supplementary file 1 [file ijms-24-05459-s001.zip › supplement figure.pdf]

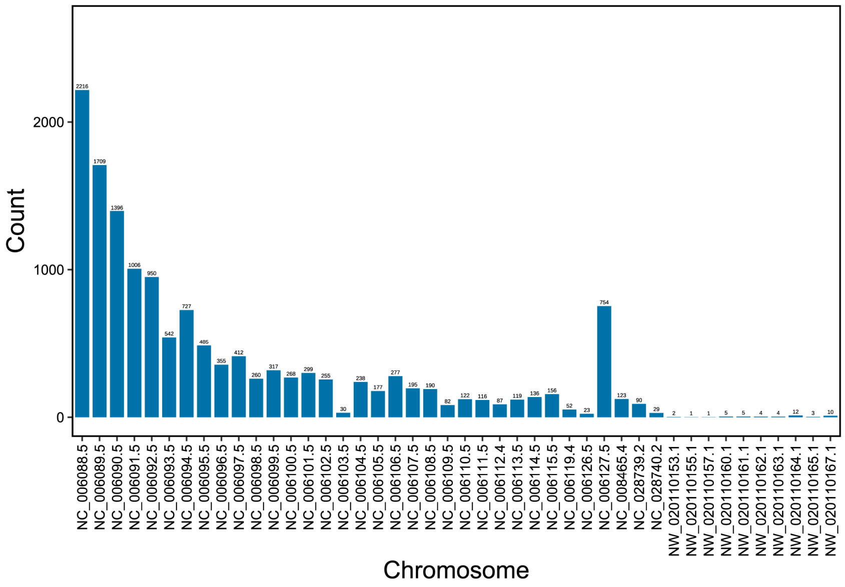

Figure S1: Chromosome distribution of circRNA transcripts identified in GM relative to DM of chicken primary myogenesis.

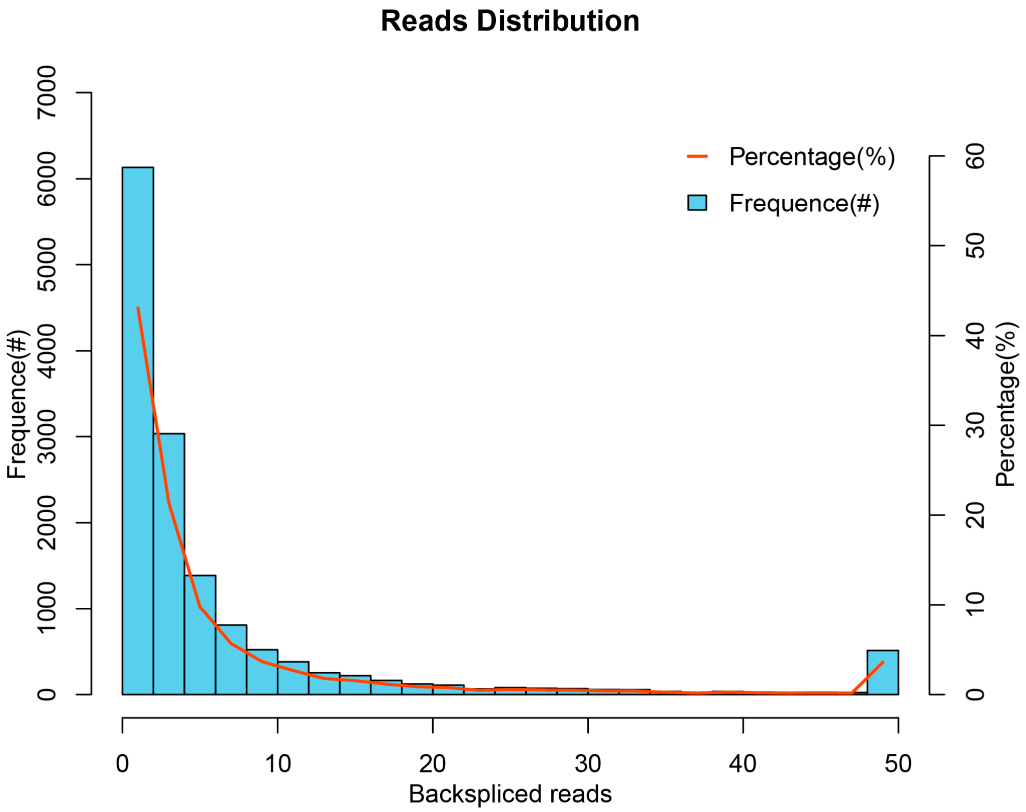

Figure S2: Backspliced reads distribution of circRNA transcripts identified in GM relative to DM of chicken primary myogenesis.

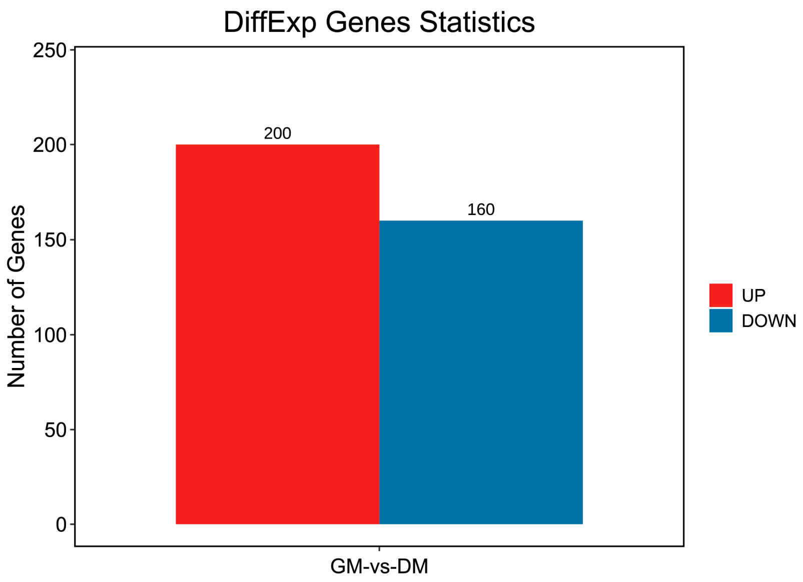

Figure S3: Backspliced reads distribution of circRNA transcripts identified in GM relative to DM of chicken primary myogenesis.

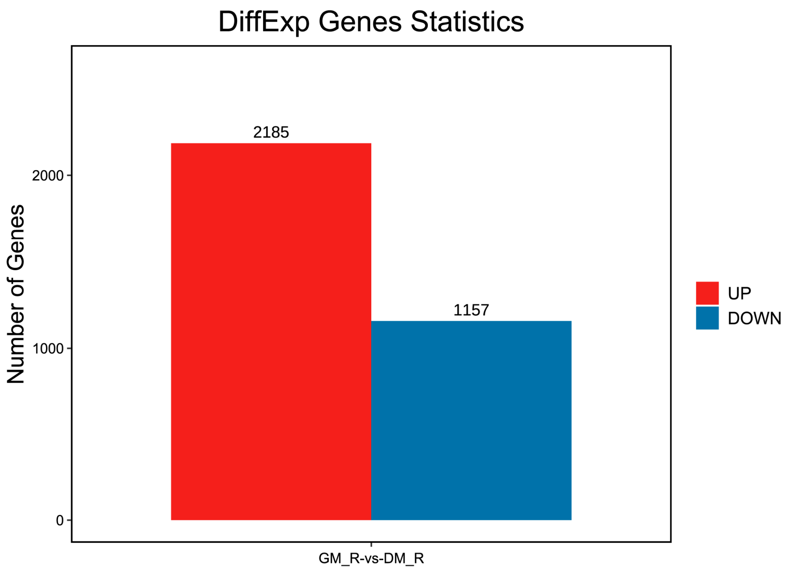

Figure S4:Differentiated-expressed genes identified in GM relative to DM of chicken primary myogenesis by using ribo-seq.
