# Supplementary material for: CircPLXNA2 Affects the Proliferation and Apoptosis of Myoblast through circPLXNA2/gga-miR-12207-5P/MDM4 Axis
Source: Int J Mol Sci. 2023 Mar 13;24(6):5459. doi: 10.3390/ijms24065459 (PMC10049439; doi:10.3390/ijms24065459)
Supplement: Supplementary file 1 [file ijms-24-05459-s001.zip › table S3.pdf]

Table S3. Information of sequences of pmir-GLO in this study

Name	3′UTR Sequences (5′–3′)	Size (bp)	Application
Pmir-GLO-*circPLXNA2*-WT	AACCATGTGAGGGA<br>CGTTGCTGGGGCACA<br>GCTCGGCCGTTGCCA<br>GGATGGATCGGAGG<br>AGGACCTGCCCTTGG<br>GCACTGCGCTCCGAC<br>GGCGATGGCAGGTC<br>AGCATCTCTGCTCTG<br>CCTCCTCCTGGCATC<br>GCTCTCCTGGAGCGC<br>ATCCGGCAGTGCCTC<br>ATTCAGCACTTTCCA<br>TTCTGAGCACCGGGA<br>CTGGACTTTCAACCA<br>CCTGACCG	215bp	Vector construction
Pmir-GLO-*circPLXNA2*-MUT	AACCATGTGAGGGA<br>CGTTGCTGGGGCACA<br>GCTCGGCCGTTGCCA<br>GGATGGATCGGAGG<br>AGGACCTGCCCTTGG<br>GCACTGCGCTCCGAC<br>GGCGATCCGTCGTCA<br>CGTTCTGACCTGACG<br>GAGCTCCTGGCATCG<br>CTCTCCTGGAGCGCA<br>TCCGGCAGTGCCTCA<br>TTCAGCACTTTCCATT<br>CTGAGCACCGGGAC<br>TGGACTTTCAACCAC<br>CTGACCG	215bp	Vector construction
Pmir-GLO-*MDM4*-WT	GGCACTGGCTTGTAA<br>TATTTCCTTTGCAGTG<br>CCATCAGATGTTGGC<br>CAAACGGTGCTATTA<br>GTTTCCTTTTCCACAT<br>GTGCTTTTCGTTGGA<br>GGTGGGTGTTGTGAG	181bp	Vector construction

	CCTCATGTTCTCTGA<br>AATCATCCCTGGCAT<br>CTCATTGTGCATAAC<br>AGCTCTGTCTCTTAA<br>CTGTGGTTTTCCTA		
Pmir-GLO-*MDM4*-MUT	GGCACTGGCTTGTAA<br>TATTTCCTTTGCAGTG<br>CCATCAGATGTTGGC<br>CAAACGGTGCTATTA<br>GTTTCCTTTTCCACA<br>ACTCGAAAAGGTTG<br>GAGGACGCACAACA<br>CAGCCTCATGTTCTC<br>TGAAATCATCCCTGG<br>CATCTCATTGTGCAT<br>AACAGCTCTGTCTCT<br>TAACTGTGGTTTTCCT<br>A	181bp	Vector construction

Note: Green letters represent wild-type binding sites, red letters represent mutant binding sites.
